#  Recurrence of uterine tissue residues after laparoscopic hysterectomy or myomectomy.

**DOI:** 10.12669/pjms.305.4509

**Published:** 2014

**Authors:** Cunjian Yi, Li Li, Xiaowen Wang, Xiangqiong Liu

**Affiliations:** 1 *Cunjian Yi, MD, PhD, Department of Obstetrics and Gynecology, First Affiliated Hospital, Yangtze University, Jingzhou, Hubei Province 434000, P.R. China.*; 2 *Li Li, MD, Department of Obstetrics and Gynecology, First Affiliated Hospital, Yangtze University, Jingzhou, Hubei Province 434000, P.R. China.*; 3 *Xiaowen Wang, MD, Department of Obstetrics and Gynecology, First Affiliated Hospital, Yangtze University, Jingzhou, Hubei Province 434000, P.R. China.*; 4 *Xiangqiong Liu, MD, Department of Obstetrics and Gynecology, First Affiliated Hospital, Yangtze University, Jingzhou, Hubei Province 434000, P.R. China.*

**Keywords:** Tissue residues, Laparoscopic myomectomy, Laparoscopic hysterectomy; Laparoscopic complications

## Abstract

***Objective: ***To report a new complication after laparoscopic surgery i.e recurrence of endometrium and leiomyoma fragments from uterine tissue residues after laparoscopic hysterectomy or laparoscopic myomectomy.

***Methods***: This study was carried out on three patients with the recurrence of endometrium or leiomyoma fragments from tissue residues after laparoscopic hysterectomy or laparoscopic myomectomy in the First Affiliated Hospital, Yangtze University, China. We also explored the possible reasons and corresponding preventative strategies.

***Results:*** Small residues of endometrium and leiomyoma fragments could implant into normal tissue anywhere in the peritoneal cavity after laparoscopic myomectomy or laparoscopic hysterectomy.

***Conclusion:*** These cases emphasize the importance of removing every single fragment to prevent the recurrence of endometrium and leiomyoma from tissue residues.

## INTRODUCTION

Laparoscopy has become a mainstay of gynecological surgery. With the advent of electromechanical morcellator, laparoscopic myomectomy and laparoscopic hysterectomy are practical alternatives for traditional surgical management of uterine fibroids. These procedures are now much more feasible and have become a common minimally invasive surgery in gynecology. Due to the use of electromechanical morcellator, the removal of large, dense specimens, such as leiomyoma fragments is not through vagina and abdominal incision, so the operation is more convenient and minimally invasive. 

Along with the extensive application of this approach, the complications started to appear. Literature reports that the incidence of postoperative complications is 1.65-4%, including fever, bladder injury, hemorrhage, and intestinal injury, trocar site hernia, etc.^1^^,^^2^ In the process of removing uterine and myoma tissues, small myoma or endometrial tissue fragments might remain in the abdominal cavity because of the limitation of operative skills and laparoscopic technique, which results in the recurrence after surgery and causes a new late postoperative complication.

## CASE REPORTS

This study was approved by the Institutional Review Board of the First Affiliated Hospital, Yangtze University, China. Three patents were admitted into the hospital between June, 2006 and February, 2012. The patients’ information is as follows:

***Patient 1:*** A 46-year-old woman was found to have a pelvic mass for 10 days during her routine gynecological examination without any other complaints and then admitted into hospital. Her records indicated that 5 years ago she underwent a laparoscopic subtotal hysterectomy due to leiomyomata in other hospital. On pelvic examination, vulva and vagina were normal and showed a closed smooth-appearing cervix, uterus was not palpable but a complex irregular mass with clear borders was found in the right lateral pelvic. The mass was about 5cm in diameter, immobility and no tenderness. Her vital signs were stable, and blood & urine analysis at laboratory examination was normal, The patient’s CA-125 serum level was 12 U/mL and CA199 serum level was 19 U/mL, transvaginal ultrasonography revealed a heterogeneous right-sided complex pelvic mass with the size of 5.6 ×4.6 × 3.8cm, which was mainly cystic with some internal echogenicity. Laparoscopy was performed under general anesthesia, an irregular mass was found densely adherent in the cul-de-sac to the cervix stump, posterior surface of the broad ligament, and to the right-lateral pelvic side-wall. The size was 5.0 × 5.0 × 4.0cm. After dissection from peritoneum, the right uterine adnexa was exposed in normal appearance and below the mass, the mass was just below and behind the peritoneum. After opening the lateral retroperitoneal space, the ureter was identified. The mass was separated from the ureter and then itwas completely isolated. After removing the mass, the cystic part of mass contained the mucinous substance in brownish color, and the appearance of solid part of mass was similar to myoma. The specimen was examined by rapid frozen pathology, and the results showed the presence of endometrial tissue and leiomyoma.

***Patient 2:*** A 36-year-old woman was admitted into hospital because of sudden left lower quadrant pain for 30 minutes after sexual intercourse. This patient underwent laparoscopic myomectomy in other hospital 2 years ago. Gynecological examination: normal external genitalia and vagina, cervical mild erosion, no contact bleeding, Cervical excitation test was positive and posterior fornix was full. The uterus was felt to be enlarged and mobile, and a large size of 3.0 cm cystic mass that was soft on palpation, smooth and somewhat fixed in the left –side pelvis was noted. Obvious tenderness in the bilateral adnexa of uterus, 5ml non-clotting blood was collected via culdocentesis. She had a negative urinalysis. Serum beta HCG was negative. White blood cell count, platelets, hematocrit and creatinine were 13,800/μl, 225,000/μl, 28% and 0.8 mg/dl, respectively. Transvaginal ultrasonography detected a heterogeneous mass (4.5 × 3.6 cm) in the left uterine adnexa and mild ascities in the pelvic cavity. The laparoscopy was performed because of hemoperitoneum. During surgery left ovary showed sign of recent rupture (yellowish tissue of corpus luteum). A myoma excrescence at a size of 2.5 × 1.5 × 2.0 cm was observed at the left trocar incision site of previous laparoscopy in the abdominal wall, which had smooth surface in dumbbell shape. After dissection, the tissue was verified as the myometrial tissue. Her histopathology report was leiomyoma.

***Patient 3:*** A 45- year-old woman was admitted into the department of general surgery because of acute appendicitis. She underwent laparoscopic hysterectomy in other hospital because of leiomyoma two years ago. Under laparoscopy, bilateral uterus adnexa were normal and the cervical stump was smooth. The left uterosacral ligament protruded outward, and a size of 4.0 × 3.0 × 3.0 cm solid mass was observed, it was buried under retroperitoneum with smooth border and immobile, adherent to the left uterosacral ligament. After exposing the left ureter and openning the mass capsule, the mass was isolated from the left utero-sacral ligament. The specimen was sent for histopathology. The pelvic mass was reported as leiomyoma.

## DISCUSSION

Laparoscopic hysterectomy and myomectomy are well-known minimally invasive surgical procedures. Laparoscopic surgery offers reduced post-operative care, shorter hospital stay, more favorable cost-benefit ratios, with a considerably shorter convalescence and a consequent earlier return to normal activities. Although this surgery has many advantages, it also has some complications. The complexity of the surgery significantly influences the complication rate. Laparoscopic complications include injuries to the vessels, bowel, urinary tract and incisional hernia.^3^ Since the introduction of laparoscopic morcellation procedures, few studies have found some complications.^4^^,^^5^ They found that morcellation increases the possibility of leaving behind small residues of endometrium & leiomyoma, which can implant into normal tissue anywhere in the peritoneal cavity.^6^^-^8 Diagnosis of the recurrence of myomas is often incidental at the time of abdominal surgery for other diseases; however, there are case reports in which the recurrence of myomas causes symptoms.^9^^,^^10^

So it is essential for the surgeon to avoid falling pieces during morcellation and effort should be made to remove all pieces to prevent their recurrence. However, the visual blind area and defects of subjective operation will result in the occurrence of tissue residues. For example, in patient 1, laparoscopy revealed iatrogenic recurrence of myoma residue and endometriosis implants in the pelvis because the woman underwent a laparoscopic subtotal hysterectomy for a leiomyoma uterus. The uterus was amputated and removed from the abdominal cavity with an electric morcellator. When they separated adnexa, they opened up broad ligaments and spillage, so the endometrial and myoma tissues might have been left in the retroperitoneal space during uterine morcellation and could grow into an endometriosis lesion and myoma, forming a heterogeneous mass under the conditions of sufficient blood supply and effect of ovarian hormones. In patient 2, this is a report of uterine leiomyoma particle growing in an abdominal wall incision after laparoscopic retrieval, suggesting inadvertent implantation at the site of removal through the trocar sleeve. As for patient 3, it might be caused by the inappropriate laparoscopic operation. The view of laparoscopic operation is different from that of traditional operation. The laparoscopic operation has only visual sense but no touch sense; it can’t touch the deep lesions. It didn’t find the small myoma below the ligation line when ligating the cervix, so the myoma grew into the lesion as mentioned in the patient 3.

According to the scattered cases for the occurrence of residual uterus tissue, the hospital and gynecologists who are using or plan to use the laparoscopic operation should pay more attention to this issue. It is necessary to train the gynecologists to master the skills in the laparoscopic surgery so that they are familiar with the microscopic vision and to use all equipments during operation. The gynecologists should have high responsibility to remove the complete tissue, and to check all visions for any missing tissue after operation. Before operation, the use of ultrasonography and even magnetic resonance imaging or computed tomography scans may be beneficial in these cases in identifying an unanticipated mass. During operation, doctors should carefully check the whole abdominal cavity for any missing lesion, especially the upper abdomen and retroperitoneal spaces which are often obscured by other organs and can be difficult to access. And it is also important that the tip of the morcellator be in view at all times. Doctors should follow standard operation procedure, and the trocar should be removed with microcopy body with the assistance of microscopy. If doctors can follow these points, the occurrence of residual uterus residue can be reduced to a great extent.

## Authors’ contributions:

**CY, LL and XW** performed operations and wrote the paper draft; **CY and XL** conceived, supervised the operations and revised the paper. All authors read and approved the final manuscript.

## Notes

***Source of funding:*** Supported by Hubei Provincial Outstanding Medical Academic Leader program.
